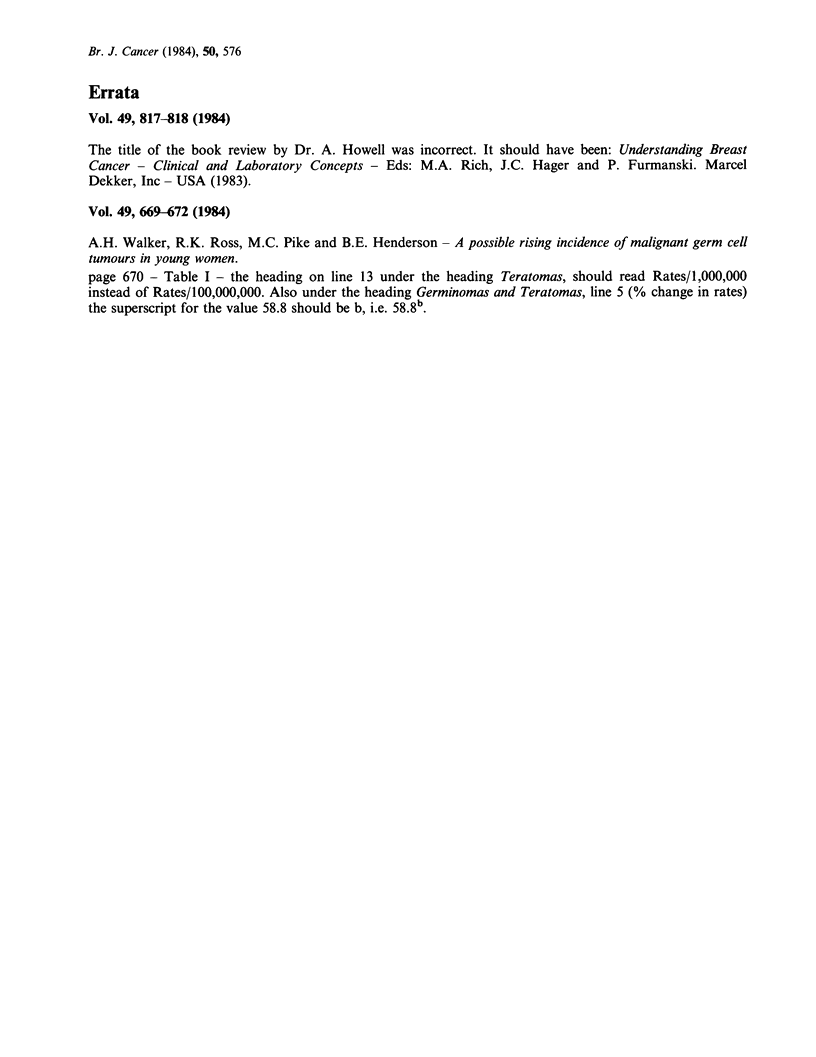# Errata

**Published:** 1984-10

**Authors:** 


*Br. J. Cancer* (1984), **50**, 576

### Errata

**Vol. 49, 817–818 (1984)**

The title of the book review by Dr. A. Howell was incorrect. It should have been: *Understanding Breast Cancer – Clinical and Laboratory Concepts* – Eds: M.A. Rich, J.C. Hager and P. Furmanski. Marcel Dekker, Inc – USA (1983).

**Vol. 49, 669–672 (1984)**

A.H. Walker, R.K. Ross, M.C. Pike and B.E. Henderson – *A possible rising incidence of malignant germ cell tumours in young women.*

page 670 – Table I – the heading on line 13 under the heading *Teratomas*, should read Rates/1,000,000 instead of Rates/100,000,000. Also under the heading *Germinomas and Teratomas*, line 5 (% change in rates) the superscript for the value 58.8 should be b, i.e. $58.8^{b}$.